# Prevalence of anaemia and associated factors among patients with type 2 diabetes mellitus in the Ho municipality in Ghana

**DOI:** 10.4314/gmj.v59i3.7

**Published:** 2025-09

**Authors:** Sylvester Y Lokpo, Daniel Yarboye, Chrisbella T Nkansah, Samuel Ametepe, Francis A Ussher, Michael Appiah, Precious K Kwadzokpui, Esther N Adejumo, Christian Obirikorang, Abigail Ibrahim, James Osei-Yeboah

**Affiliations:** 1 Department of Medical Laboratory Sciences, School of Allied Health Sciences, University of Health and Allied Sciences, Ho, Ghana; 2 Department of Medical Laboratory Science, Faculty of Health and Allied Sciences, Koforidua Technical University, Koforidua, Ghana; 3 Department of Medical Laboratory Sciences, Accra Technical University, Accra, Ghana; 4 Labortory Department, Ho Teaching Hospital, Ministry of Health, Ho, Ghana; 5 Department of Medical Laboratory Science, School of Public and Allied Health, Babcock University, Ilishan-Remo, Ogun State, Nigeria; 6 Department of Molecular Medicine, School of Medicine and Dentistry, Kwame Nkrumah University of Science and Technology, Kumasi, Ghana; 7 Nursing and Midwifery Training College, Odumase-Krobo, Ghana; 8 School of Public Health, Kwame Nkrumah University of Science and Technology, Kumasi, Ghana

**Keywords:** Prevalence, Anaemia, Type 2 Diabetes Mellitus, Body Mass Index, Platelets

## Abstract

**Objective:**

This study aimed to determine the prevalence of anaemia and associated factors among type 2 diabetic patients.

**Design:**

This research utilised a hospital-based cross-sectional study design.

**Setting:**

The research was conducted at the Diabetic Clinic of Ho Municipal Hospital.

**Participants:**

The study involved 180 type 2 diabetic patients, aged 20 years or older, who had been on anti-diabetic medications for a minimum of three months before the study. Premenopausal women who had not menstruated in the two weeks before recruitment were also included in the study. Participants were excluded if they were receiving haematinics, had undergone a blood transfusion in the preceding month, were undergoing treatment for malaria or helminthiasis, or had any other chronic complications such as renal failure, liver disease, or stroke. Individuals with type 1 diabetes and pregnant women were excluded from the study.

**Results:**

Approximately a quarter [44 (24.4%)] of the study population had anaemia, with a slight male preponderance [15(25.0%)]. Mild and moderate anaemia were 31 (70.5%) and 13(29.5%), respectively. Microcytic hypochromic anaemia [16 (36.4%)] was the most frequent morphological type of anaemia, followed by normocytic normochromic anaemia [12(27.3%)]. High BMI and low platelet counts were independently associated with reduced odds of developing anaemia in patients with type 2 diabetes mellitus.

**Conclusion:**

Anaemia is a common finding in patients with type 2 diabetes mellitus in the Ho municipality. Mild anaemia and microcytic hypochromic anaemia were predominant among the anaemic patients. High BMI and low platelet count were significant predictors of reduced probability of anaemia.

**Funding:**

None declared

## Introduction

Anaemia is a condition that refers to a decrease in haemoglobin concentration that varies by age, gender, and geography. In anaemia, red blood cells have reduced oxygen-carrying capacity and cannot meet the body's physiological requirements.^1^ Thus, the World Health Organisation (WHO) defines anaemia as haemoglobin levels less than 12.0 g/dL in women and less than 13.0 g/dL in men.^2^

Recent reports suggest that anaemia affects approximately 22.8% of people of all ages worldwide, contributing to about 58.6 million years of life with disability, with sub-Saharan Africa recording the highest burden of anaemia.^3^ Anaemia also contributes to poor cognitive and motor development in children and work capacity in adults, which has direct or indirect implications for the country's economic development.^4^

Diabetes mellitus is a metabolic disorder with several etiologies characterised by prolonged high blood glucose levels resulting in disturbances in carbohydrate, fat, and protein metabolism.^5^ Diabetes mellitus is a highly disabling disease that has the potential to cause blindness, amputations, functional incapacity and autonomy impairment, kidney disease, cardiovascular and brain complications, anaemia, as well as reduced quality of life.^6^ The International Diabetes Federation (IDF) estimates that about 463 million persons aged 20 to 79 years worldwide have diabetes mellitus. At this rate, this figure is projected to rise to 643 million by 2030 and 783 million by 2045, with almost three-quarters of adults with diabetes mellitus residing in low- and middle-income countries.^7^ The pathogenesis of anaemia in diabetes mellitus is multifactorial, including factors such as inflammation, hormonal changes, nutritional deficiencies, concomitant autoimmune diseases, and the use of certain drugs in individuals with underlying kidney disease.^8^ Numerous studies indicate that anaemia is higher in patients with diabetes mellitus than in those without the condition.^9^-^11^ Anaemia is a risk factor for cardiovascular events that is independent of other factors. This is particularly true when patients with diabetes mellitus experience a precipitous decline in renal function and a heightened need for renal replacement therapy.^11^ Unfortunately, the burden of anaemia is not well characterised, and recent reports have indicated that approximately 25% of diabetic patients with anaemia in sub-Saharan Africa remain undiagnosed. Moreover, the condition is associated with an elevated risk of morbidity and mortality.^1^,^9^ Despite this, most current research on anaemia has been skewed toward certain aspects of the population, such as children, adolescents, and pregnant women.^11^-^13^

Meanwhile, the prevalence of diabetes mellitus among adults from a community-based study in the Ho municipality remains high (6.9%) 14 compared to the national statistics (2.7%)^15^, although lower than the current global statistics (11.1%).^16^ Moreover, previous studies among diabetic patients have largely focused on elucidating the risk factors of diabetes mellitus and the assessment of metabolic syndrome.^17^,^18^ There is yet a study dedicated to understanding anaemia burden among patients with diabetes mellitus. Therefore, this study aims to determine the prevalence, degree, and factors associated with anaemia among patients with type 2 diabetes mellitus in the Ho municipality. The findings of this study will inform polices aimed at providing holistic care in the management of diabetes mellitus in the Ho municipality.

## Methods

### Study design and study site description

This research utilised a hospital-based cross-sectional study design. The research was conducted at the Diabetic Clinic of Ho Municipal Hospital. The hospital is situated within the Ho municipality of the Volta Region, Ghana. It currently operates a 150-bed capacity and receives referral cases from health centres and Community-based Health Planning and Services (CHPS) compounds within the city and neighbouring Republic of Togo. Its services include outpatient services, inpatient services, maternity care, antenatal clinics, diabetic clinics, herbal clinics, and counselling services. The diabetic clinic operates from Monday to Friday, with approximately 20 patients visiting daily for clinical management.

### Study population and sampling technique

The study population included patients with type 2 diabetes mellitus undergoing clinical management at the Ho Municipal Hospital. The participants were recruited using a convenient sampling technique. Those who were 20 years or older, had been on anti-diabetic medications for a minimum of three (3) months before the study, those who were not taking haematinic, had not received a blood transfusion in the previous month, were not undergoing treatment for malaria or helminthiasis, and had no chronic conditions such as sickle cell disease and thalassaemia or complications including kidney disease, liver disease, or stroke. Additionally, premenopausal women who were not menstruating or had not had their period in two weeks at the time of recruitment were also included in this study.

### Sample size determination

The Raosoft Online Sample Calculator was used to determine the sample size for this study. A minimum sample size of 178 was calculated from 330 registered patients with type 2 diabetes mellitus at the Diabetic Clinic, using a 95% confidence interval and a 5.0% margin of error. However, a total of 180 participants were recruited for this study.

### Data collection

This study employed a semi-structured questionnaire to obtain demographic information (age, gender, marital status, educational background, and occupation), lifestyle data (number of hours spent working per day, alcohol intake, and anthropometry), and clinical information (diabetes duration and type of diabetes treatment).

### Blood pressure and anthropometric measurements

A mercury sphygmomanometer and a stethoscope were used to measure the blood pressure after participants had rested for at least 5 minutes. Height was measured with participants standing upright and their feet together, without shoes, using a stadiometer. The value of the height was imputed into a bioelectrical impedance device, where the BMI was calculated based on an inbuilt algorithm. Hip circumference was measured as the widest part around the buttocks, and waist circumference as the lowest part between the iliac crest and rib cage. Waist-to-hip ratio was calculated as the waist circumference divided by the hip circumference. The waist-to-height ratio was calculated by dividing the waist circumference by the height.

### Haematological and biochemical measurements

After an overnight fast (10-12 hours), approximately 5 millilitres (ml) of venous blood samples were obtained from the antecubital vein. Two millilitres (2 ml) were dispensed into an Ethylenediaminetetraacetic acid (EDTA) tube and inverted eight times to ensure thorough mixing of the blood with the anticoagulant. Three millilitres (3 ml) were dispensed into a gel separator tube and centrifuged at 3000 revolutions per minute for five minutes at room temperature to obtain the serum. Whole blood was used to quantify haemoglobin levels, platelet counts, and red blood cell indices (mean corpuscular volume and mean corpuscular haemoglobin concentration) employing XN 550i Haematology Analyser. The serum obtained was used to measure creatinine levels on a Selectra Pro Chemistry autoanalyser using ELI-Tech-based reagents at the Clinical Chemistry Unit. The estimated glomerular filtration rate (eGFR) was computed using the CKD-EPI formula.

### Estimated glomerular filtration rate (eGFR) calculations

The Glomerular Filtration Rate (eGFR) was estimated using the formula: GFR = 141 × min (Scr × 0.0113 / κ, 1) ^α^ × max (Scr × 0.0113 / κ, 1) ^−1.209^ × 0.993^Age^ × 1.018 [for females] × 1.159 [for Black individuals]. In this formula, Scr denotes serum creatinine measured in mg/dL, κ is defined as 0.7 for females and 0.9 for males, α is -0.329 for females and -0.411 for males, “min” refers to the smaller value between Scr/κ or 1, and “max” refers to the larger value between Scr/κ or 1.^19^

### Definition of central obesity and glycemic control

Central obesity was defined as a waist circumference for men, ≥ 94 cm, and women: ≥ 80 cm according to the harmonised criteria. In contrast, glycaemic control was defined as the FBG value falling within the fasting range (3.9- 5.6 mmol/L) proposed by the WHO.^20^

### Statistical analysis

Data were generated in a Microsoft Excel 2016 spreadsheet, cleaned, and subsequently exported to the Statistical Package for Social Sciences (SPSS) version 26 software for analysis. Categorical data were expressed as frequencies and corresponding proportions. Binary and multivariate logistic regression analyses were performed to identify factors associated with anaemia. A p-value of <0.05 was considered significant.

**Approval of research protocol:** Ethical clearance was granted by the University of Health and Allied Sciences Research Ethics Committee (UHAS REC), with protocol identification number UHAS-REC A.11 [16] 21-22. Written informed consent was obtained from all participants recruited into this study.

## Results

Table 1 presents the demographic, lifestyle, and clinical characteristics of study participants. One hundred and eighty (180) participants were recruited for this study. The majority were above 50 years old (57.2%), were females (66.7%), and 68.3% were married; 57.2% had their basic education, while 73.3% worked in the informal sector. Approximately 34.4% worked for more than 8 hours a day, whereas 72.2% were either overweight or obese, and 56.1% had central obesity. Uncontrolled blood pressure and uncontrolled glucose levels were 16.7% and 84.4%, respectively. At the time of this study, the majority (45.0%) had been diagnosed with diabetes for less than 5 years, and 66.7% had been on combined oral and insulin treatments. Reduced renal function was 18.9% and 58.3% based on serum creatinine levels and eGFR of <90 mL/min/1.73m^2^, respectively. Low platelet count was observed in 28.3% while self-report of alcohol intake was 18.3%.

**Table 1:** Demographic, lifestyle, and clinical characteristics of study participants

Parameters	Frequency (%)	Percent
Total	180 (100)	100.0
**Age (years)**		
≤50	77 (42.8)	42.8
>50	103 (57.2)	57.2
**Gender**		
Male	60 (33.3)	33.3
Female	120 (66.7)	66.7
**Marital status**		
Single	57 (31.7)	31.7
Married	123 (68.3)	68.3
**Educational Background**		
None	16 (8.9)	8.9
Basic	103 (57.2)	57.2
Secondary	27 (15.0)	15.0
Tertiary	34 (18.9)	18.9
**Occupation**		
Formal	36 (20.0)	20.0
Informal	132 (73.3)	73.3
None	12 (6.7)	6.7
**Hours of work/day**		
≤8 hours	118 (65.6)	65.6
>8 hours	62 (34.4)	34.4
**BMI (kg/m^2^)**		
Normal	50 (27.8)	27.8
Overweight	59 (32.8)	32.8
Obese	71 (39.4)	39.4
**Central Obesity**		
No	79 (43.9)	43.9
Yes	101 (56.1)	56.1
**Blood Pressure**		
Normal	150 (83.3)	83.3
High	30 (16.7)	16.7
**Diabetes Duration (years)**		
<5	81 (45.0)	45.0
5-10	69 (38.3)	38.3
>10	30 (16.7)	16.7
**Diabetes Treatment**		
Oral Only	60 (33.3)	33.3
Oral + Insulin	120 (66.7)	66.7
**Glycemic Control**		
Yes	28 (15.6)	15.6
No	152 (84.4)	84.4
**Creatinine (mmol/L)**		
Normal	146 (81.1)	81.1
High	34 (18.9)	18.9
**eGFR (mL/min/1.73 m^2^)**		
<60	25 (13.9)	13.9
60-90	80 (44.4)	44.4
>90	75 (41.7)	41.7
**Platelet Count**		
Normal	129 (71.7)	71.7
Low	51 (28.3)	28.3
**Alcohol Intake (current)**		
No	147 (81.7)	81.7
Yes	33 (18.3)	18.3

Data is presented as the frequency and corresponding proportion. BMI-Body Mass Index, eGFR-Estimated Glomerular Filtration Rate

**Figure 1:**
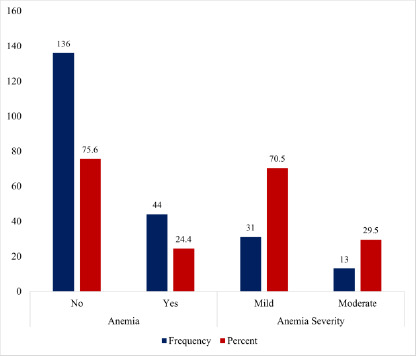
Prevalence of anaemia and distribution of anaemia severity among study participants

**Figure 2:**
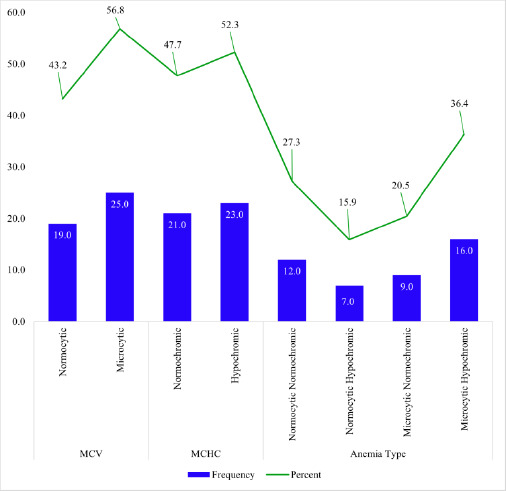
Distribution of mean corpuscular volume (MCV), mean corpuscular haemoglobin concentration (MCHC), and anaemia type among study participants

As shown in Figure 1, anaemia was present in 24.4% of the total study participants. In terms of the severity of anaemia, 70.5% had mild anaemia, whereas 29.5% presented with moderate anaemia. Among the anaemic patients in Figure 2, microcytosis and hypochromasia were observed in 56.8% and 52.3% of participants, respectively. Further analysis revealed that 27.3% presented with normocytic-normochromic anaemia, whereas 36.4% presented with microcytic-hypochromic anaemia.

From the univariate and multivariate logistic regression analyses presented in Table 2, high BMI (AOR: 0.45, 95% CI: 0.21-0.95; p = 0.036) and low platelet count (AOR: 0.26, 95% CI: 0.09-0.73; p = 0.010) were significantly associated with a reduced risk of anaemia.

**Table 2:** Binary and multivariate logistic regression analysis of variables associated with anaemia among patients with type 2 diabetes

Anaemia						
Variables	Yes	No	cOR (95% CI)	p-value	aOR (95% CI)	p-value
**Age (years)**						
**≤50**	21(27.3)	56(72.7)	1			
**>50**	23(22.3)	80(77.7)	0.77(0.39-1.52)	0.446		
**Gender**						
**Male**	15(25.0)	45(75)	1			
**Female**	29(24.2)	91(75.8)	0.96(0.47-1.96)	0.902		
**Marital status**						
**Single**	13(24.1)	41(75.9)	1			
**Married**	31(24.6)	95(75.4)	1.02(0.49-2.17)	0.940		
**Educational Background**						
**Yes**	5(31.3)	11(68.8)	1			
**No**	39(23.8)	125(76.2)	1.46(0.48-4.45)	0.509		
**Employment status**						
**Employed**	9(25.0)	27(75.0)	1			
**Unemployed**	35(24.3)	109(75.7)	1.04(0.45-2.42)	0.931		
**Hours of work/day**						
**≤8 hours**	32(27.1)	86(72.9)	1			
**>8 hours**	12(19.4)	50(80.6)	0.65(0.30-1.36)	0.251		
**Alcohol intake (current)**						
**No**	39(26.5)	108(73.5)	1			
**Yes**	5(15.2)	28(84.8)	0.49(0.18-1.37)	0.176		
**Diabetes Duration (years)**						
**≤5**	20(20.8)	76(79.2)	1		1	
**>5**	24(28.6)	60(71.4)	1.52(0.77-3.01)	0.230	1.32(0.64-2.71)	0.454
**Diabetic Treatment**						
**Oral Only**	14(23.3)	46(76.7)	1			
**Oral + Insulin**	30(25.0)	90(75)	1.10(0.53-2.27)	0.806		
**BMI (kg/m^2^)**						
**Normal**	18(36.0)	32(64)	1		1	
**High**	26(20.0)	104(80.0)	0.44(0.22-0.91)	**0.027**	0.45(0.21-0.95)	**0.036**
**Central Obesity**						
**No**	21(26.6)	58(73.4)	1			
**Yes**	23(22.8)	78(77.2)	0.81(0.41-1.61)	0.555		
**BP Control**						
**No**	8(26.7)	22(73.3)	1.15(0.47-2.81)	0.757		
**Yes**	36(24.0)	114(76.0)	1			
**Glycaemic Control**						
**Yes**	8(28.6)	20(71.4)	1			
**No**	36(23.7)	116(76.3)	0.78(0.32-1.91)	0.581		
**Platelet Count**						
**Normal**	39(30.2)	90(69.8)	1		1	
**Low**	5(9.8)	46(90.2)	0.25(0.09-0.68)	**0.007**	0.26(0.09-0.73)	**0.010**
**Creatinine (mmol/L)**						
**Normal**	33(22.6)	113(77.4)	1		1	
**High**	11(32.4)	23(67.6)	1.64(0.72-3.71)	0.236	1.36(0.58-3.23)	0.480
**eGFR (mL/min/1.73 m^2^)**						
**≤90**	25(23.8)	80(76.2)	0.92(0.46-1.83)	0.815		
**>90**	19(25.3)	56(74.7)	1			

BMI-Body Mass Index, WHR-Waste to Hip ratio, BP-Blood Pressure; eGFR-Estimated Glomerular Filtration Rate; cOR-Crude Odds Ratio; aOR-Adjusted Odds Ratio; CI-Confidence Interval

However, age, gender, marital status, educational background, employment status, alcohol intake, duration of diabetes, type of diabetes treatment, central obesity, BP control, glycaemic control, high creatinine level, and low eGFR were not significantly associated with anaemia.

## Discussion

In type 2 diabetes mellitus, anaemia is a progressively recognised entity of public health significance that can lead to serious morbidity and mortality.^21^ Anaemia contributes to the development and progression of cardiovascular disease, chronic renal disease, and diabetic retinopathy, as well as other diabetic complications.^22^ Recent studies have shown that anaemia is common in patients with type 2 diabetes mellitus, with rates ranging from 20.1% to 65%.^5^,^23^,^24^ However, the burden of anaemia has not been well characterised among patients with diabetes mellitus in sub-Saharan Africa.^24^ To our knowledge, no study had previously evaluated the burden of anaemia among diabetic patients in the Volta Region. Hence, we conducted the current study to determine the prevalence and ascertain associated factors of anaemia in patients with type 2 diabetes mellitus in the Ho municipality.

Here, we present a summary of the key findings from our study; approximately a quarter [44 (24.4%)] of the study population had anaemia, with a slight male preponderance [15(25.0%)]. Mild and moderate anaemia were 31 (70.5%) and 13(29.5%), respectively. Microcytic hypochromic anaemia [16 (36.4%)] was the most frequent morphological type of anaemia followed by normocytic normochromic anaemia [12(27.3%)]. High BMI and low platelet counts were independently associated with reduced odds of developing anaemia in patients with type 2 diabetes mellitus.

The prevalence of anaemia in this study is consistent with findings from Ethiopia (24.8%) 25 and the United Arab Emirates (29.7%).^26^ However, our finding contrasts with Yorke et al.^27^ who reported a higher prevalence of anaemia (53.1%) among patients with type 2 diabetes mellitus in the Greater Accra Region. Differences in population characteristics, including the length of diabetes diagnosis (median: 5 vs 9.4 years) and possibly nutritional characteristics (not measured in this study) could account for the disparity in the anaemia burden between our study and that of Yorke et al.^27^ Several potential pathological pathways have been proposed to contribute to the development of anaemia in patients with diabetes mellitus. These include low erythropoietin levels, iron deficiency, subclinical inflammation associated with chronic kidney disease, loss of transferrin, and increased destruction of red blood cells due to morphological disorders caused by diabetes mellitus.^28^ Furthermore, anti-diabetic medications such as metformin are known to cause vitamin B12 malabsorption, leading to its deficiency with the resulting clinical symptoms of anaemia.^21^ Meanwhile, the male preponderance to anaemia in this study is also consistent with the findings of El Saied 23, Teshome et al. 29, Yorke et al. 27, and Kaushik et al. 30, but contrary to Manoj & Navneet 21, and Rathod et al.^31^

Regarding the severity of anaemia, the prevalence of mild anaemia is consistent with a similar study in Ethiopia (84%).^5^ The mild and moderate forms of anaemia are frequent features of anaemia due to chronic diseases such as diabetes mellitus that may be linked to the expression and progression of diabetic micro- and macroangiopathies.^32^ Moreover, the predominance of a microcytic and hypochromic blood picture observed in our study could be due to nutritional deficiencies leading to reduced iron levels and poor glycaemic control, as exhibited by most of the study participants. Iron deficiency resulting from whatever cause as well as the toxicity to red blood cells due to prolonged hyperglycaemia have been associated with microcytic hypochromic anaemia.^33^ Although a previous study has posited that microcytic hypochromic anaemia could suggest anaemia of renal origin in patients with diabetes mellitus,^34^ we did not find anaemia to be significantly influenced by biomarkers of renal dysfunction, probably due to the relatively low proportion of anaemia recorded in our study. Notwithstanding, our findings correspond with previous reports of Kaushik et al.^30^

Our finding that a high BMI level significantly reduces the likelihood of anaemia [AOR: 0.45 (0.21-0.95), p = 0.036] is counterintuitive but consistent with the findings of previous studies. Katarzyna et al.^35^, and Yu et al.^36^ found that being overweight or obese was associated with a lower risk of anaemia among Chinese and Colombian women, . More recently, Asmae et al.^37^ found a significant inverse correlation between BMI and haemoglobin levels in patients with type 2 diabetes mellitus at a referral centre in Oujda-Angad, Morocco. The reason for our finding cannot be inferred directly from this study, but it is argued that low levels of iron and vitamin C intake may be partly responsible for the incidence of anaemia among people with overweight and obesity.^36^ This might also be the case in our study, hence, the lower risk of anaemia.

Similarly, our finding that participants with low platelet counts had a significantly lower risk of anaemia [AOR: 0.26 (0.09-0.73) p=0.010] was contrary to the findings of several studies that reported a significant association between low platelet counts and higher risk of anaemia 38-40 which were largely attributed to iron deficiency. Again, it is difficult to offer any plausible explanation for our findings; hence, we suggest further studies to clarify this.

This study has some limitations worth mentioning; hence, the interpretation of the findings should be made in the following contexts. The study design employed was cross-sectional, which limited the ability to detect any causal effect. There is also the potential for recall bias, coupled with the unavailability of comprehensive data on dietary characteristics, which limits our understanding of how these factors may influence our study findings.

## Conclusion

Anaemia is a common finding in patients with type 2 diabetes in the Ho municipality. Mild anaemia and microcytic hypochromic anaemia were more predominant among the anaemic patients. High BMI and low platelet count were independently associated with a reduced probability of anaemia.
